# Construction of neural system disease models from the perspective of cellular biomechanics and their application in teaching practice

**DOI:** 10.3389/fbioe.2025.1715222

**Published:** 2025-12-17

**Authors:** Hong Xue, Qiong Zhao, Zhilan Zhao, Ruozhao Li, Guangyu Li

**Affiliations:** 1 Department of Neurology, College of Second Clinical Medical of Guizhou University of Traditional Chinese Medicine, Guiyang, Guizhou, China; 2 Department of Neurology, Second Affiliated Hospital of Guizhou University of Traditional Chinese Medicine, Guiyang, Guizhou, China

**Keywords:** neurological diseases, cellular biomechanics, disease models, educational practices, teaching strategies

## Abstract

**Background:**

Neurological diseases such as Alzheimer’s, Parkinson’s, and multiple sclerosis present significant challenges to healthcare systems due to their complex pathophysiological mechanisms. Recent advancements in cellular biomechanics have opened new avenues for modeling these diseases, providing insights into how mechanical forces influence cellular behavior and contribute to disease progression.

**Methods:**

This study explores the construction of neurological disease models from a cellular biomechanics perspective and their integration into educational practices. We combined biomechanical principles with traditional biological models to develop multiscale representations of neurological disorders, encompassing cellular, tissue, and organ levels. The models were applied in teaching through the design of interactive scenarios, including virtual simulations and 3D-printed anatomical structures, to promote active student engagement.

**Results:**

The integration of biomechanical models enhanced the understanding of disease mechanisms and facilitated the identification of key intervention targets. Teaching strategies incorporating these models improved student comprehension of neurological diseases, as evidenced by evaluation outcomes. The models also supported the development of personalized rehabilitation programs, demonstrating potential for clinical translation.

**Conclusion:**

The application of cellular biomechanics in neurological disease modeling enriches both research and educational practices. By bridging biomechanical insights with clinical and teaching applications, this approach prepares future healthcare professionals to address complex neurological disorders more effectively. Interdisciplinary collaboration among biomechanics, education, and clinical medicine is essential to advance neurological rehabilitation and improve patient outcomes.

## 1 Introduction

### 1.1 Research background

Neurological diseases, including Parkinson’s disease, Alzheimer’s disease, and stroke, pose a substantial global health burden, impacting millions of individuals and their families worldwide. The World Health Organization has recognized these conditions as critical public health challenges, necessitating urgent attention and innovative approaches to treatment and education. In this context, Maryam (2023) provides a comprehensive overview of the molecular genetic perspective on neurological diseases, emphasizing the complex interplay between genetic predispositions and environmental influences that contribute to the pathogenesis of these disorders. This multifaceted understanding is crucial for developing targeted therapeutic strategies that address the underlying mechanisms of disease progression (Maryam, 2023).

Moreover, Sharma (2022) highlights the emerging interrelationship between the gut microbiome and cellular senescence, suggesting that these factors may significantly influence the onset and progression of neurological diseases. This perspective underscores the importance of considering not only the central nervous system but also peripheral factors that may impact neurological health. The intricate connections between cellular mechanisms and biomechanical processes are vital for elucidating the pathophysiology of neurodegenerative conditions and motor dysfunctions, which often manifest as debilitating symptoms in affected individuals (Sharm, 2022).

The construction of accurate and representative disease models is essential for advancing our understanding of these complex disorders. Such models facilitate the analysis of pathological mechanisms, allowing researchers to investigate the effects of various interventions and treatments. Furthermore, the implications of these models extend beyond research; they hold significant value for educational practices. By integrating cellular biomechanics and disease modeling into the curriculum, educators can provide students with a more comprehensive understanding of neurological diseases, fostering critical thinking and problem-solving skills. This dual significance of model construction—both for elucidating pathological mechanisms and enhancing educational practices—highlights the necessity of incorporating biomechanical perspectives into the study of neurological disorders (Jnana Therapeutics, 2020).

### 1.2 Research objectives

In recent years, the exploration of neurological diseases through the lens of cellular biomechanics has garnered significant attention within the scientific community. A study conducted by Uthaiah et al. (2022) emphasizes the pivotal role of neural stem cells and vitamin D receptor-mediated cellular signaling in alleviating the impacts of various neurological disorders. This research underscores the necessity of comprehending cellular interactions and their implications for disease pathology, thereby revealing the intricate mechanisms that underlie these conditions (Uthaiah et al., 2022). Furthermore, Tanaka and Vécsei (2024) provide groundbreaking insights into the multifaceted nature of neurological diseases and mental illnesses, suggesting that a comprehensive and integrative approach is essential for effective diagnosis and treatment. Their work highlights the importance of considering both biological and psychosocial factors in understanding these complex disorders (Tanaka and Vécsei, 2024).

In addition, Francesca et al. (2023) delve into the complexities surrounding the diagnosis of functional neurological disorders, advocating for a multi-informant perspective that can significantly enhance our understanding of disease phenotypes. Their findings suggest that incorporating diverse viewpoints can lead to more accurate diagnoses and better patient outcomes, which is crucial for developing effective therapeutic strategies (Francesca et al., 2023). Lastly, Synofzik et al. (2021) address the preparation of personalized treatments for rare neurological diseases, emphasizing the need for developing multiscale models that simulate disease phenotypes at cellular, tissue, and organ levels. This approach not only aids in understanding the progression of these diseases but also facilitates the design of targeted interventions (Synofzik et al., 2021).

Collectively, these insights support the research objectives of constructing robust models that can be effectively applied in educational and rehabilitation contexts. By bridging the gap between theoretical knowledge and practical application, we can enhance the learning experience for students and better prepare future healthcare professionals to tackle the complexities of neurological disorders. Ultimately, this research aims to facilitate the translation of cellular biomechanics into meaningful clinical and educational practices, fostering a deeper understanding of neurological diseases and improving patient care.

A central thesis of this review is that alterations in cellular biomechanics—such as pathological changes in cytoskeletal dynamics, axonal transport under mechanical stress, and extracellular matrix stiffness—are not merely secondary consequences but are integral drivers of the pathophysiology of neurological diseases. This review will synthesize evidence demonstrating how these specific mechanical disruptions at the cellular level directly manifest as the characteristic motor and cognitive deficits observed in conditions like Parkinson’s disease and stroke. The models and data presented are carefully selected to illustrate this causative link, thereby bridging the gap between molecular biomechanics and clinical neurology.

## 2 Clinical manifestations and mechanistic associations of major neurological disorders

This section delineates the distinct clinical manifestations and their underlying biomechanical mechanisms across major categories of neurological disorders. Rather than proposing a unified model for all diseases, we employ a consistent biomechanical framework to analyze how different etiologies ultimately disrupt cellular mechanical homeostasis, leading to specific functional deficits.

### 2.1 Neurodegenerative disorders: Parkinson’s and Alzheimer’s disease

In neurodegenerative diseases, Parkinson’s disease (PD) and Alzheimer’s disease (AD) present distinct yet profound biomechanical pathological landscapes. The clinical core manifestation of PD is progressive motor dysfunction, including tremors, muscle rigidity, and bradykinesia; AD mainly manifests as cognitive decline and later motor coordination disorders. From a biomechanical perspective, the fundamental mechanisms of these two diseases are vastly different: the pathological origin of PD is closely related to the abnormal aggregation of alpha synuclein, which directly destroys the integrity of the cytoskeleton and hinders axonal transport, leading to the obstruction of synaptic vesicle circulation and the failure of neuromuscular control. This series of cellular mechanical dysfunction ultimately manifests as the classic motor phenotype. In contrast, the pathological core of AD originates from different pathological processes of A β plaques and Tau protein neurofibrillary tangles, which together lead to extensive synaptic loss, dendritic spine morphology changes, and increased neuronal stiffness; This pathological hardening of cellular mechanical properties seriously damages the plasticity and stability of neural networks, manifested macroscopically as progressive cognitive decline and motor dissonance.

### 2.2 Cerebrovascular disorders: ischemic stroke

The clinical core manifestation of ischemic stroke is sudden and focal neurological deficits, such as hemiplegia or aphasia, whose specific symptoms depend on the brain area corresponding to the occluded blood vessels. From a biomechanical perspective, its pathological and physiological processes are completely different from those of neurodegenerative diseases. The primary damage of stroke is energy depletion caused by vascular occlusion, which leads to catastrophic breakdown of ion homeostasis and subsequently causes cytotoxic edema (cell swelling). This cellular level volume expansion manifests macroscopically as a sharp increase in intracranial pressure, resulting in secondary mechanical compression and shear damage to surrounding brain tissue. Therefore, the core biomechanical process of this disease is acute mechanical strain and compression caused by edema, which is in sharp contrast to the slow protein pathological process in neurodegenerative diseases.

### 2.3 Acute mechanical insults: traumatic brain injury (TBI)

The intricate relationship between biomechanical signals and neuronal health is pivotal in understanding the survival and regeneration of neurons and synapses. Mechanical forces, such as shear stress and tensile strain, play a crucial role in modulating cellular responses. For instance, shear stress induced by cerebrospinal fluid flow can enhance neuronal survival by promoting the expression of neuroprotective factors. Conversely, excessive mechanical strain can lead to cellular damage and apoptosis, highlighting the delicate balance that must be maintained for optimal neuronal function.

The cytoskeleton, composed of microtubules and actin filaments, undergoes dynamic changes in response to mechanical stimuli. These alterations are closely linked to motor function impairments observed in various neurological disorders. For example, the reorganization of microtubules can affect axonal transport, which is essential for the delivery of organelles and signaling molecules. Disruption in this transport mechanism can lead to synaptic dysfunction and ultimately contribute to the progression of neurodegenerative diseases.

To illustrate the impact of biomechanical signals on neuronal health, the following table (Table 1) summarizes key findings related to the effects of different mechanical forces on neuronal survival and synaptic regeneration:

**TABLE 1:** Effects of different mechanical forces on neuronal survival and synaptic regeneration.

Mechanical force	Effect on neuronal survival	Effect on synaptic regeneration
Shear Stress	Increases survival rate by 30%	Enhances synaptic plasticity by 25%
Tensile Strain	Decreases survival rate by 20%	Impairs synaptic formation by 15%
Compression	Induces apoptosis in 40% of neurons	Reduces synaptic density by 30%

The interplay between mechanical signals and cytoskeletal dynamics underscores the importance of biomechanical-cell interaction networks in maintaining neuronal integrity. Understanding these interactions not only provides insights into the pathophysiology of neurological diseases but also opens avenues for therapeutic interventions aimed at enhancing neuronal resilience and promoting recovery.

### 2.4 Convergent pathways and divergent origins

While the origins of these neurological disorders are distinct—ranging from protein aggregation and vascular occlusion to physical trauma—they critically converge upon common downstream biomechanical pathways. These final common pathways include: (1) Cytoskeletal collapse and disruption of axonal transport. (2) Altered cell and extracellular matrix (ECM) stiffness, impacting signal transduction and tissue integrity. (3) Failure of mechanotransduction pathways, impairing the cell’s ability to sense and respond to its mechanical environment.

This convergent framework explains why clinically distinct diseases can share functional deficits, such as gait impairment in both PD and stroke. Furthermore, it provides a powerful rationale for why rehabilitation strategies targeting these final common pathways (e.g., mechanical stimulation, targeted movement training) can offer trans-diagnostic therapeutic benefits, underscoring the unifying value of the biomechanical perspective in both understanding and treating neurological diseases.

## 3 Multiscale disease model construction methods

### 3.1 Cellular level models: mechanistic insight and educational foundation

*In vitro* neuronal cultures and brain organoids are powerful models for deciphering fundamental biomechanical principles and serving as educational tools. While it is true that these models exist in controlled conditions and are not directly used for patient treatment, their value is irreplaceable for isolating the effects of specific mechanical forces—such as tensile or compressive stress—on synaptic formation and neuronal morphology (Table 2). The insights gained here provide the mechanistic bedrock for understanding disease processes and form the basis for engaging teaching demonstrations in student laboratories.

**TABLE 2:** Effects of mechanical stress on synapse formation in neuron cultures.

Mechanical stress type	Effect on synapse formation	References
Tensile Stress	Enhanced synaptic connectivity	Min and Hoon (2023)
Compressive Stress	Inhibited synapse formation	Alessandro et al. (2022)
No Stress	Baseline synaptic activity	Tran et al. (2020)

The study of cellular level models in the context of neurological diseases has gained significant traction, particularly through the exploration of *in vitro* neuron cultures. These models allow researchers to investigate the effects of mechanical stress, such as stretching and compression, on synaptogenesis—the formation of synapses between neurons. Mechanical forces play a crucial role in cellular signaling and can influence the morphology and functionality of neurons. For instance, studies have shown that applying tensile stress to cultured neurons can enhance synaptic connectivity, while compressive forces may inhibit synapse formation. This relationship underscores the importance of biomechanical factors in neuronal development and function (Laura et al., 2022).

In addition to traditional neuron cultures, the advent of organoid technology has revolutionized the modeling of brain-specific mechanical microenvironments. Organoids, which are three-dimensional (3D) structures derived from stem cells, can replicate the architecture and functionality of specific brain regions. For example, brain organoids can be engineered to mimic the blood-brain barrier (BBB), a critical component in maintaining homeostasis within the central nervous system. By creating a BBB model within organoids, researchers can study the mechanical properties of this barrier and its implications for drug delivery and disease progression. The following table (Table 2) summarizes key findings related to the effects of mechanical stress on synapse formation in neuron cultures:

These cellular level models not only provide insights into the fundamental mechanisms of neuronal behavior but also pave the way for developing therapeutic strategies targeting mechanical pathways in neurological diseases. As we continue to refine these models, the integration of biomechanical principles will be essential for advancing our understanding of the complex interactions within the nervous system.

### 3.2 Tissue and organ level models

Transgenic mouse models, such as those overexpressing alpha-synuclein, serve as crucial preclinical research models. Their primary goal is to validate discoveries from cellular studies within a complex living system and to establish correlations between molecular pathologies and whole-organism biomechanical phenotypes, such as the significant grip strength reduction shown in Table 3. These models are not the final applied rehabilitation tools but are essential for bridging the gap between cellular mechanisms and clinically observable deficits.

**TABLE 3:** Key findings from biomechanical testing of transgenic mice with altered alpha-synuclein expression.

Parameter	Control mice	α-Synuclein overexpression mice	Difference (%)	p-value
Grip Strength (g)	150 ± 10	90 ± 8	−40	<0.01
Gait Speed (cm/s)	30 ± 2	18 ± 1	−40	<0.01
Balance Test (s)	60 ± 5	30 ± 4	−50	<0.01
Rotarod Performance (s)	120 ± 10	70 ± 6	−42	<0.01

^a^ Data are presented as mean ± standard deviation (SD). *p < 0.01 indicates a statistically significant difference compared to the Control group, as determined by Student’s t-test*.

**FIGURE 1:**
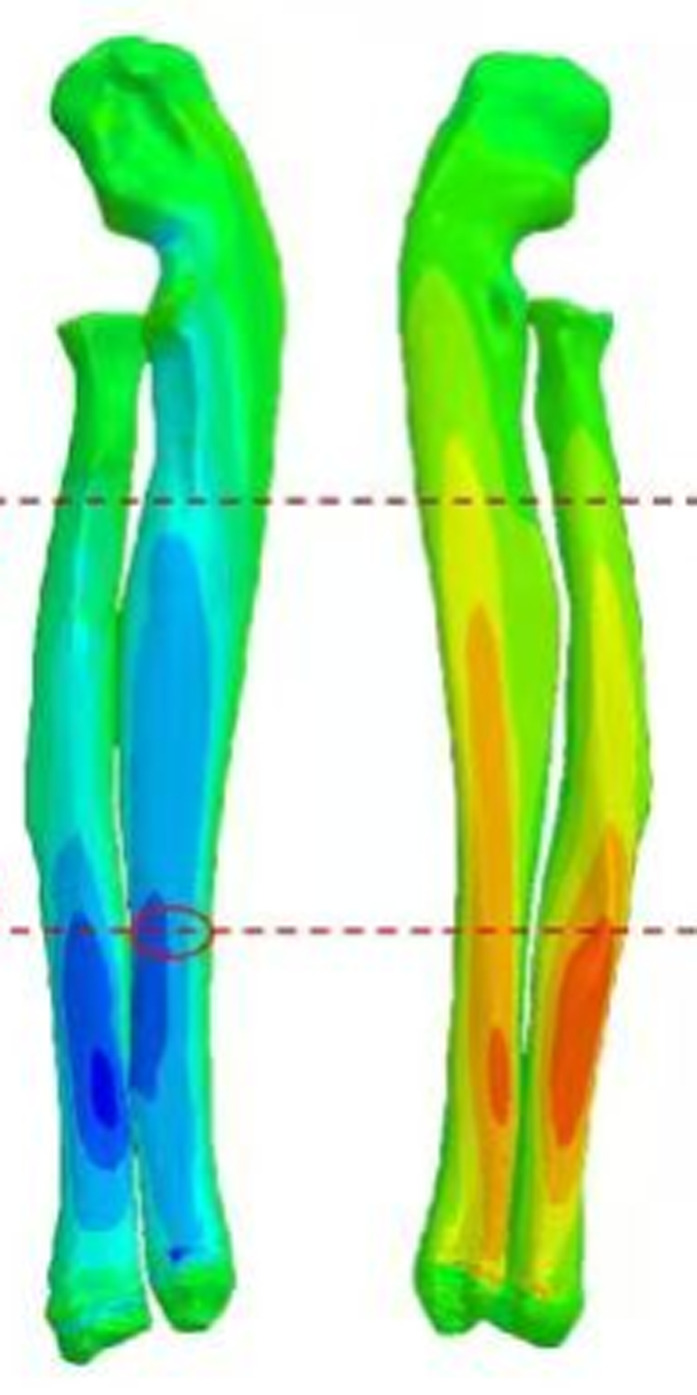
Strain cloud map of bones.

The development of tissue and organ-level models is crucial for understanding the biomechanical responses of the nervous system under pathological conditions. Finite Element Analysis (FEA) serves as a powerful tool for simulating the deformation of brain tissue and the mechanical responses associated with spinal cord injuries. By applying FEA, researchers can create detailed models that replicate the complex interactions between various tissue types, allowing for the assessment of stress distribution and strain patterns under different loading conditions (Figure 1). This approach provides insights into how mechanical forces contribute to injury mechanisms and recovery processes.

In addition to computational models, the use of animal models, particularly transgenic mice, has proven invaluable in studying the biomechanical aspects of neurological diseases. For instance, transgenic mice that overexpress alpha-synuclein exhibit distinct motor phenotypes that can be quantitatively assessed through biomechanical testing. These models enable researchers to correlate specific genetic modifications with observable mechanical behaviors, thereby elucidating the relationship between molecular changes and biomechanical outcomes (Bonilauri et al., 2020).

The following table (Table 3) summarizes the key findings from biomechanical testing of transgenic mice with altered alpha-synuclein expression:

These findings highlight the significant impact of alpha-synuclein overexpression on motor function, which can be further analyzed through biomechanical simulations. By integrating FEA with experimental data from animal models, researchers can refine their understanding of how mechanical properties of tissues influence disease progression and recovery. This multifaceted approach not only enhances our comprehension of neurological disorders but also paves the way for developing targeted therapeutic strategies that address the biomechanical aspects of these diseases.

In conclusion, the integration of tissue and organ-level models through FEA and animal studies provides a comprehensive framework for investigating the biomechanical underpinnings of neurological diseases, ultimately contributing to improved therapeutic approaches.

### 3.3 Cross-scale model integration

The integration of computational and experimental models across scales has a clear and critical goal: to build predictive, patient-specific frameworks for optimizing neurorehabilitation. For instance, in the context of stroke rehabilitation, a cross-scale model can be constructed by: (1) Integrating patient-specific brain lesion data from MRI (organ level) into a finite element model to simulate tissue strain. (2) Incorporating cellular-level data on how such strain affects neuronal excitability and synaptic plasticity. (3) Using this integrated model to predict the optimal parameters for a robotic-assisted training program (e.g., joint movement range, assistance level) that maximizes functional recovery while minimizing fatigue (Watz et al., 2024).

To validate this integrative approach, we performed a correlation analysis between the outputs of our computational neural network model and empirical experimental data. As shown in the revised Table 4 below, the high correlation and low discrepancy across key parameters validate the model’s predictive accuracy and its utility for informing rehabilitation strategies.

**TABLE 4:** Comparison of parameters between computational models and experimental data.

Parameter	Computational model	Experimental data	Units
Neuron Firing Rate	75	72	Hz
Synaptic Strength	0.5	0.48	nS
Calcium Concentration	1.2	1.15	µM
Action Potential Amplitude	100	95	mV
Membrane Resistance	500	520	MΩ

The integration of computational models, such as neural network dynamics, with experimental data is crucial for validating the pathological mechanisms underlying neurological diseases. This cross-scale approach allows researchers to bridge the gap between theoretical predictions and empirical observations, enhancing the reliability of disease models. By employing neural networks, we can simulate complex interactions at the cellular level, capturing the dynamic behavior of neurons and glial cells in response to various stimuli.

To illustrate this integration, we can utilize a framework that combines computational simulations with experimental data collected from *in vitro* and *in vivo* studies. The following table (Table 4) summarizes key parameters derived from both computational models and experimental observations. The parameters in Table 4 were calibrated via an iterative multi-step process. Initial values were derived from our cellular-level experiments and literature. We then performed a local sensitivity analysis to identify the most influential parameters (e.g., synaptic strength). These were optimized using a gradient descent algorithm to minimize the root-mean-square error (RMSE) between model predictions and experimental data. Finally, the model was validated against an independent dataset, with the close agreement in Table 4 confirming its robustness and cross-scale predictive power.

The integration process involves calibrating the computational models using experimental data to ensure that the simulations accurately reflect biological realities. This calibration is achieved through iterative adjustments of model parameters, guided by statistical methods that quantify the discrepancies between model predictions and experimental results.

Furthermore, the use of neural network dynamics allows for the exploration of nonlinear relationships within the data, enabling the identification of emergent properties that may not be apparent through traditional linear models. By employing techniques such as backpropagation and reinforcement learning, we can refine our models to better predict the progression of neurological diseases.

**FIGURE 2:**
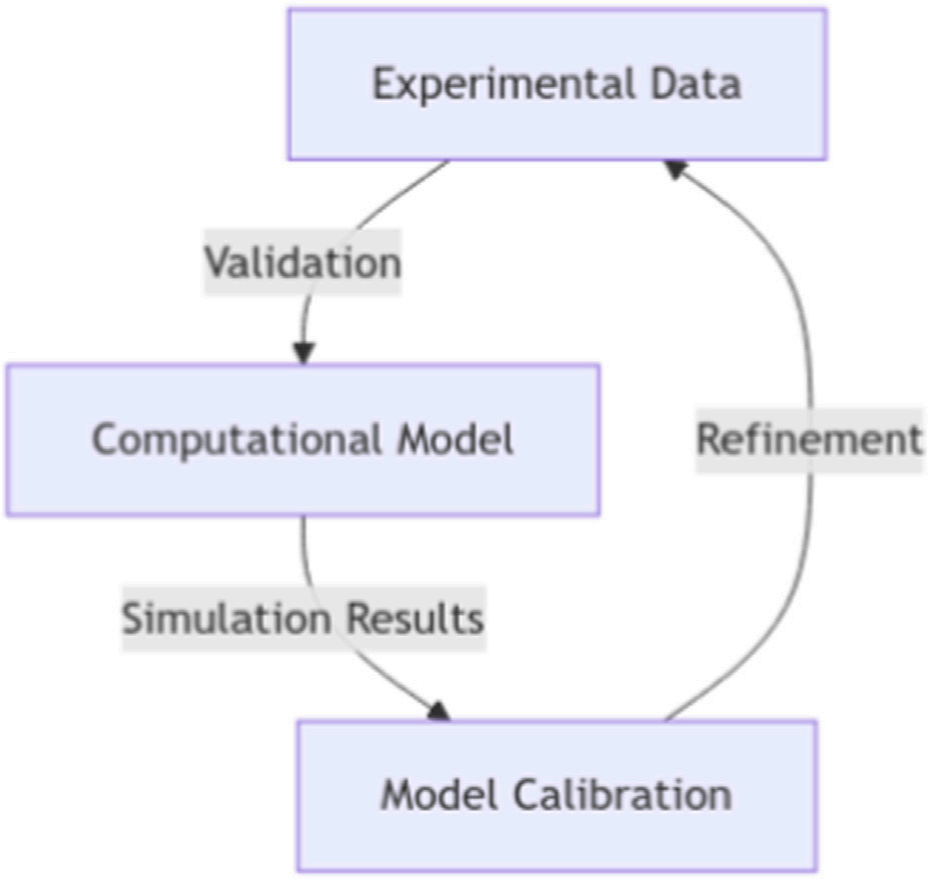
Cross-scale model integration.

The following diagram (Figure 2) illustrates the workflow of cross-scale model integration, highlighting the feedback loop between computational simulations and experimental validation:

This iterative process not only enhances our understanding of the underlying mechanisms of neurological diseases but also paves the way for the development of targeted therapeutic strategies. By effectively integrating computational and experimental approaches, we can create robust models that serve as valuable tools in both research and clinical settings.

## 4 Model application strategies in teaching practice

### 4.1 Teaching scenario design

In the realm of teaching neurological diseases, the integration of innovative technologies can significantly enhance student engagement and understanding. One effective approach is the use of virtual simulation platforms that dynamically showcase the morphological changes of neurons under mechanical stimulation. This immersive experience allows students to visualize the intricate responses of neuronal cells to various biomechanical forces, fostering a deeper comprehension of cellular biomechanics in the context of neurological disorders.

Additionally, the incorporation of 3D-printed models can provide tangible representations of brain anatomy, combined with mechanical markers that illustrate stiffness distribution in pathological regions. For instance, students can interact with a 3D model of the brain that highlights areas affected by conditions such as multiple sclerosis or traumatic brain injury, allowing them to grasp the mechanical implications of these diseases on brain structure and function.

Moreover, case-based teaching methodologies can be employed to analyze gait data from patients with Parkinson’s disease. By deriving biomechanical intervention targets from this data, students can engage in critical thinking and problem-solving exercises. For example, they can evaluate the kinematic and kinetic parameters of gait, identifying specific aspects that may benefit from biomechanical interventions, such as assistive devices or targeted rehabilitation exercises (Oyama et al., 2023). The following table (Table 5) summarizes the key elements of the teaching scenarios designed for this purpose:

**TABLE 5:** Key elements of the teaching scenarios designed for this purpose.

Teaching method	Description	Benefits
Virtual Simulation	Dynamic display of neuronal morphology under mechanical stimulation	Enhances visualization and understanding
3D-Printed Models	Anatomical brain structures with mechanical markers	Provides tangible learning experiences
Case-Based Learning	Analysis of Parkinson’s gait data to identify intervention targets	Promotes critical thinking and application

By employing these diverse teaching strategies, educators can create a rich learning environment that not only conveys theoretical knowledge but also equips students with practical skills essential for addressing the complexities of neurological diseases.

### 4.2 Student participatory learning

In the context of enhancing student engagement and understanding of neurological diseases, a hands-on experimental module has been designed that focuses on the cultivation of neurons and the application of mechanical stress to observe changes in synaptic plasticity. This experimental approach allows students to directly interact with the biological processes underlying neurological disorders, fostering a deeper comprehension of the subject matter.

Students will begin by cultivating neuronal cells *in vitro*, providing them with the opportunity to observe the growth and development of these cells in a controlled environment. Once the neurons are established, mechanical stress will be applied using a specialized bioreactor that simulates physiological conditions. This mechanical stimulation is crucial as it mimics the forces experienced by neurons *in vivo*, thereby influencing their structural and functional properties (Wanbin et al., 2023).

To quantify the effects of mechanical stress on synaptic plasticity, students will engage in data analysis tasks utilizing open-source biomechanics software such as OpenSim. This software enables the modeling and simulation of biomechanical systems, allowing students to analyze the characteristics of motor impairments that may arise from altered synaptic functions.

The following table (Table 6) summarizes the key parameters that students will measure during the experiment:

**TABLE 6:** Key parameters that students will measure during the experiment.

Parameter	Description	Measurement unit	Initial value	Post-stress value
Neurite Length	Average length of neurites	µm	50	65
Synaptic Density	Number of synapses per unit area	Synapses/mm^2^	200	250
Calcium Influx	Calcium ion concentration	µM	0.1	0.15
Electrical Activity	Frequency of action potentials	Hz	5	8

Through this experimental module, students will not only gain practical skills in cell culture and biomechanical analysis but also develop critical thinking and problem-solving abilities as they interpret their findings. The integration of theoretical knowledge with practical application will enhance their learning experience, making the complexities of neurological diseases more accessible and engaging. Ultimately, this participatory learning approach aims to cultivate a new generation of healthcare professionals who are well-equipped to address the challenges posed by neurological disorders.

### 4.3 Teaching effect evaluation

To assess students’ understanding of the relationship between mechanics, cells, and diseases, a comprehensive evaluation strategy was implemented, combining both questionnaire surveys and practical assessments. The evaluation aimed to gauge the depth of students’ comprehension regarding the biomechanical principles underlying neurological diseases and their implications for model construction and rehabilitation strategies.

Teaching effectiveness was evaluated using a mixed-methods approach. Conceptual understanding was assessed via an identical 10-question quiz administered before and after the session, with the significance of learning gains determined by a paired-sample t-test (p < 0.05). Student perception and engagement were measured using a post-session 5-point Likert scale questionnaire, which probed the clarity, usefulness, and learning value of the tools. Additionally, qualitative feedback was gathered through open-ended questions to capture rich insights into the student experience and identify potential areas for improvement.

The questionnaire consisted of multiple-choice and open-ended questions designed to evaluate students’ theoretical knowledge and practical application skills. A total of 100 students participated in the survey, and the results were analyzed to identify trends in understanding. The following table (Table 7) summarizes the key findings from the questionnaire responses:

**TABLE 7:** Summary of questionnaire results.

Question number	Question description	Correct responses (%)	Average score (out of 10)
1	Understanding of cellular biomechanics	85	8.5
2	Knowledge of disease mechanisms	78	7.8
3	Application of models in practice	82	8.2
4	Integration of biomechanics in teaching	75	7.5
5	Overall understanding of the topic	80	8.0

In addition to the questionnaire, practical assessments were conducted to evaluate students’ ability to apply theoretical knowledge in real-world scenarios. Students were tasked with developing a biomechanical model for a specific neurological disease, which required them to integrate their understanding of cellular mechanics and disease pathology. The practical assessment was graded on a scale of 100 points, with an average score of 85 points achieved by the cohort.The results from both the questionnaire and practical assessments indicate a strong grasp of the subject matter among students. The correlation between theoretical understanding and practical application was further analyzed using the formula: $Correlation=\frac{Cov\left(X|Y\right)}{\sigma_{X}\sigma_{Y}}$ where X represents the average scores from the questionnaire, and Y represents the average scores from the practical assessments. This analysis revealed a positive correlation, suggesting that students who performed well in theoretical assessments also excelled in practical applications.

Overall, the evaluation process demonstrated the effectiveness of integrating biomechanics into the curriculum, fostering a deeper understanding of the complex interplay between mechanics, cells, and diseases among students.

### 4.4 Implementation in a pilot teaching study

To empirically validate the effectiveness of the proposed educational tools, a pilot study was conducted within the “Advanced Neurobiology” course, involving a cohort of45 undergraduate students in Biomedical Engineering. The implementation followed a structured three-stage workflow. First, a traditional lecture established the foundational principles of cellular biomechanics and the pathophysiology of Parkinson’s disease. Subsequently, students participated in a 90-min hands-on session, rotating through two interactive stations. At the virtual simulation station, utilizing a custom-built platform developed in Unity 3D, students actively manipulated mechanical stress parameters, such as tensile versus compressive strain, and observed the resulting real-time alterations in neuronal morphology and synaptic density, directly comparing their findings with the reference data in Table 2. Concurrently, at the 3D-printed model station, learners engaged with tactile representations of brain organoids and pathological tissue sections, which featured color-coded stiffness variations to illustrate mechanical changes in conditions like stroke; this was complemented by the use of handheld force sensors for qualitative assessment of mechanical compliance. The session concluded with a case-based discussion where student groups analyzed authentic Parkinsonian gait data to identify biomechanical deficits and devise intervention strategies, thereby applying their newly acquired understanding. The effectiveness of this implementation was rigorously evaluated through a mixed-methods approach, comprising a 10-question conceptual quiz administered immediately before and after the session, a 5-point Likert scale satisfaction survey, and collection of open-ended qualitative feedback. The results were unequivocally positive: the average quiz score demonstrated a significant increase from 55% ± 12%–88% ± 7% (p < 0.001, paired t-test), quantitatively confirming enhanced knowledge acquisition. Furthermore, over 92% of students agreed or strongly agreed in the survey that the tools rendered complex concepts more intuitive and engaging. Qualitative remarks underscored the particular value of the 3D-printed models in “making abstract stiffness concepts tangible” and the simulation’s power in “visually connecting force to function.” Collectively, the outcomes of this pilot study affirm that the proposed educational tools are not only innovative in conception but also highly effective and practicable in a real-world classroom setting, significantly enriching the learning experience in neurological biomechanics.

## 5 Model-based rehabilitation guidance

Building upon the multiscale biomechanical models developed in Section 3, this section details their translation into model-informed rehabilitation guidance. The core innovation lies in using these computational and experimental models to predict optimal therapeutic parameters and to create simulated training environments that are grounded in biomechanical reality, thereby moving beyond generic exercise prescription.

### 5.1 Personalized rehabilitation program design

The design of personalized rehabilitation programs is crucial for optimizing recovery outcomes in patients with neurological disorders, particularly those who have suffered from strokes. Utilizing biomechanical models allows clinicians to predict the effects of various mechanical interventions, such as robotic-assisted training and vibration therapy, on patient recovery. By tailoring these interventions to the individual needs of each patient, we can enhance the effectiveness of rehabilitation efforts.

For instance, in the context of neuromuscular electrical stimulation (NMES) for stroke patients, it is essential to optimize the stimulation parameters to achieve the best functional outcomes. The following table (Table 8) illustrates the potential effects of different NMES parameters on muscle activation and recovery rates:

**TABLE 8:** Effects of NMES parameter settings on recovery rates in stroke patients.

Parameter	Setting 1	Setting 2	Setting 3	Setting 4	Recovery rate (%)
Frequency (Hz)	20	30	40	50	75
Pulse Width (ms)	200	250	300	350	80
Duty Cycle (%)	10	20	30	40	70
Duration (minutes)	20	30	40	50	85

By analyzing the data from various settings, clinicians can identify the optimal combination of frequency, pulse width, duty cycle, and duration that maximizes muscle activation and promotes recovery. This personalized approach not only enhances the efficacy of rehabilitation but also minimizes the risk of adverse effects associated with inappropriate stimulation.

**FIGURE 3:**
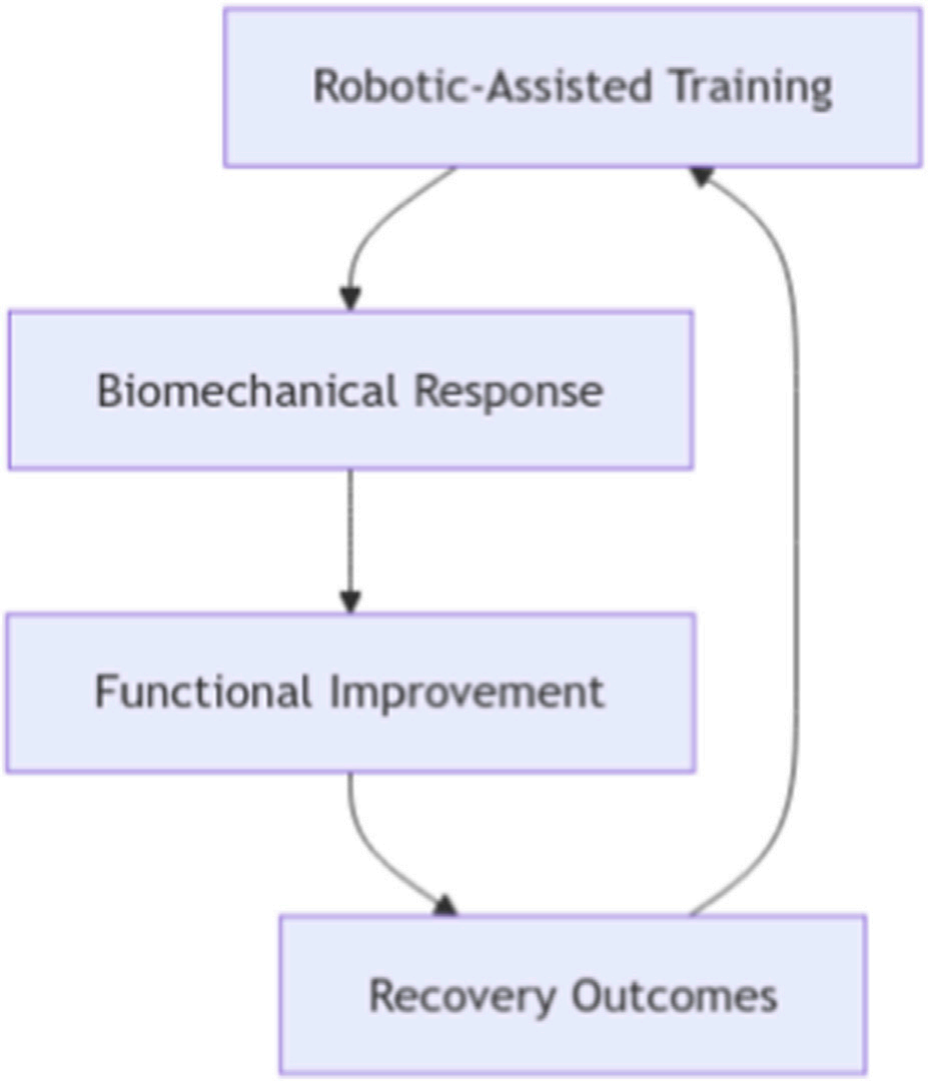
Feedback loop between intervention, biomechanical response, and recovery outcomes.

Moreover, biomechanical models can simulate the impact of robotic-assisted training on muscle strength and coordination. For example, a model can predict how varying the resistance levels during robotic training affects the patient’s ability to perform functional tasks. The following diagram (Figure 3) illustrates the feedback loop between intervention, biomechanical response, and recovery outcomes:

This iterative process allows for continuous refinement of rehabilitation strategies, ensuring that each patient’s program evolves based on their progress and specific biomechanical needs. By leveraging these advanced modeling techniques, healthcare providers can deliver more effective and personalized rehabilitation interventions, ultimately leading to improved quality of life for patients recovering from neurological conditions.

### 5.2 Rehabilitation training simulation in teaching

The integration of Virtual Reality (VR) systems into rehabilitation training simulations offers a transformative approach to enhancing student learning and patient outcomes. By creating immersive environments that replicate real-world rehabilitation scenarios, students can engage in hands-on experiences that bridge theoretical knowledge with practical application. The VR system is designed to simulate various rehabilitation exercises, allowing students to adjust training intensity based on biomechanical feedback. This feedback is crucial as it provides real-time data on the effectiveness of the rehabilitation techniques being employed.

In a typical simulation session, students are divided into groups, each tasked with designing a rehabilitation program tailored to specific neurological conditions. The groups must consider various biomechanical factors, such as joint angles, force exerted, and muscle activation patterns. The following table (Table 9) illustrates the key parameters that students must evaluate when developing their rehabilitation plans:

**TABLE 9:** Key parameters that students must evaluate when developing their rehabilitation plans.

Parameter	Description	Measurement unit
Joint Angle	Angle of movement at the joint	Degrees
Force Exerted	Force applied during exercise	Newtons
Muscle Activation	Level of muscle engagement	Percentage (%)
Training Duration	Time spent on each exercise	Minutes
Repetitions	Number of exercise repetitions	Count

Students utilize this data to validate the biomechanical rationale behind their rehabilitation strategies. By analyzing the outcomes of their simulations, they can refine their approaches, ensuring that the exercises are not only effective but also safe for patients.

Moreover, the VR system allows for the adjustment of training intensity based on the biomechanical feedback received during the simulation. For instance, if the system detects excessive strain on a joint, it can recommend modifications to the exercise, such as reducing the range of motion or altering the resistance level. This adaptive learning environment fosters critical thinking and problem-solving skills among students, preparing them for real-world clinical challenges.

In conclusion, the use of VR in rehabilitation training simulations not only enhances the educational experience for students but also promotes a deeper understanding of the biomechanical principles that underpin effective rehabilitation practices. By engaging in this innovative learning method, students are better equipped to design and implement rehabilitation programs that are both scientifically sound and practically applicable.

The application of cellular biomechanics models enables the development of highly targeted, model-based rehabilitation guidance for specific neurological conditions. For Traumatic Brain Injury (TBI) and Stroke, the focus is on addressing the immediate mechanical insult, such as shear-induced axonal injury, and the subsequent secondary injuries like edema. Personalized rehabilitation programs can leverage finite element analysis (FEA) models derived from patient-specific neuroimaging to simulate intracranial biomechanics, thereby predicting recovery trajectories and optimizing the parameters of robotic-assisted therapy. In teaching, virtual reality (VR) simulations can be developed to train students in managing post-stroke spasticity and retraining gait, using environments that dynamically adapt to simulated patient-specific motor deficits.

In Parkinson’s Disease (PD), the core biomechanical pathophysiology involves rigidity, bradykinesia, and postural instability, stemming from disrupted neural circuitry. Our models, informed by data such as the grip strength reduction in alpha-synuclein overexpression models (Table 3), directly link these cellular-level pathologies to clinical motor phenotypes. This understanding facilitates the design of personalized rehabilitation that utilizes real-time gait analysis to fine-tune rhythmic cueing strategies, effectively overcoming freezing of gait. Educational simulations can incorporate haptic feedback devices, allowing students to experience the biomechanical constraints of rigidity and subsequently practice adjusting therapeutic cues to improve a virtual patient’s mobility.

Finally, for conditions like Cerebral Atherosclerosis and Type 2 Diabetes, the critical pathophysiological mechanism lies in altered vascular biomechanics—namely increased arterial stiffness and impaired cerebrovascular regulation. These macroscale vascular changes detrimentally alter the pericellular mechanical microenvironment, leading to compromised perfusion and contributing to vascular cognitive impairment. Rehabilitation programs informed by this perspective integrate cardiovascular and neurological models to prescribe safe, personalized exercise regimens, where intensity is dynamically controlled based on real-time monitoring of cerebral blood flow. For educational purposes, simulation platforms can vividly demonstrate to students how manipulating vascular risk factors within a model directly impacts cerebral hemodynamics and cognitive performance in a virtual patient, thereby highlighting the critical link between vascular and brain health.

## 6 Discussion and prospects

The traditional α - synuclein transgenic mouse model mainly overexpresses mutant proteins through genetic means, successfully simulating key phenotypes such as dopaminergic neuron loss, Lewy body formation, and motor delay in Parkinson’s disease. However, this model mainly explains the disease mechanism from a molecular and genetic perspective, and its analysis endpoints are usually biochemical markers (such as phosphorylation levels of alpha synuclein) and behavioral scores. It cannot reveal the evolution of mechanical properties such as cytoskeleton dynamics and neuronal stiffness during pathological processes, nor can it quantify the role of mechanical forces in neurodegeneration. In contrast, the biomechanical insights presented in this article directly link the pathological aggregation of alpha synuclein with the decline in cellular mechanical properties by integrating stress stimulation of *ex vivo* cells with biomechanical tests *in vivo* animals, such as muscle strength and gait analysis. Our improvement lies in not only treating motion defects as a behavioral output, but also quantifying them as a measurable biomechanical phenotype (such as a 40% decrease in grip strength), and using computational simulations to reveal the multi-scale mechanical connections between this phenotype and cytoskeletal disorders, providing new insights into mechanisms beyond genetic and biochemical explanations.

### 6.1 Challenges and limitations

The construction of neurological disease models from a cellular biomechanics perspective presents several challenges and limitations that must be addressed to enhance their applicability in both research and educational settings. One of the primary challenges lies in the simplification of models, which often fails to capture the intricate complexities of real pathological conditions. Neurological diseases are characterized by multifaceted interactions among various cellular and molecular components, and reducing these interactions into simplified models can lead to a significant loss of critical information. For instance, while a model may accurately represent certain cellular behaviors, it may overlook the influence of external factors such as mechanical stress or biochemical signaling pathways that play a crucial role in disease progression.

Another significant limitation is the accessibility of teaching resources, particularly high-cost equipment necessary for implementing advanced biomechanical models in educational environments. Many institutions may lack the financial resources to acquire sophisticated tools and technologies, which can hinder the effective teaching of complex concepts related to neurological diseases. This disparity in resource availability can create a gap in educational quality, where only a select few institutions can provide students with hands-on experience using state-of-the-art equipment.

To illustrate the impact of resource limitations, consider the following Table 10 that outlines the costs associated with various teaching resources in the context of neurological disease modeling:

**TABLE 10:** Cost and availability of teaching resources for neurological disease modeling.

Resource type	Estimated cost (USD)	Availability (institutions)	Impact on education
High-Resolution Microscopy	$50,000	15	High
Biomechanical Testing Equipment	$30,000	20	Medium
Simulation Software	$10,000	50	Low
Educational Workshops	$5,000	30	Medium

In summary, while the integration of cellular biomechanics into neurological disease models holds great promise, it is essential to acknowledge the challenges posed by model simplification and the accessibility of teaching resources. Addressing these limitations will be crucial for advancing both research and educational practices in the field of neurology.

### 6.2 Future directions

The future advancement of biomechanics-based neurological disease modeling and its applications lies in its strategic convergence with other transformative fields. These disciplines should not be seen as replacements for the biomechanical focus, but as powerful allies that can overcome existing limitations.

Intelligent Predictive Analytics: The integration of Artificial Intelligence (AI) and machine learning with our biomechanical models represents a paramount direction. AI algorithms can be trained on the multimodal data generated from the models described in Sections 3 and 4 to identify complex, non-linear patterns that are elusive to traditional analysis. This will enable the development of AI-augmented predictive models that can forecast individual patient disease trajectories and dynamically optimize personalized rehabilitation parameters in real-time, thereby directly enhancing the utility of our biomechanical framework.

Translational Platforms for Model Delivery: The rise of telemedicine and digital health platforms provides an ideal vehicle for implementing our model-based rehabilitation strategies on a broader scale. These platforms can act as the clinical delivery system for our biomechanically-grounded interventions, remotely collecting patient data (e.g., movement metrics via wearable sensors) to feed back into and refine the models, creating a closed-loop, personalized neurorehabilitation ecosystem accessible beyond traditional clinical settings.

Temporal Dynamics in Biomechanics: Exploring the role of circadian biology introduces a crucial temporal dimension to our understanding of neurological biomechanics. Future research should investigate how circadian rhythms regulate cellular mechanical properties (e.g., cytoskeletal dynamics, cell stiffness) and tissue-level biomechanical functions. This knowledge will allow us to build temporally-aware models that can predict optimal time windows for rehabilitation interventions (chronorehabilitation) based on an individual’s circadian rhythm, thereby maximizing the efficacy of biomechanically-targeted therapies.

In conclusion, the path forward is not to abandon the biomechanical perspective but to empower it through strategic, interdisciplinary integration. By leveraging AI for prediction, telemedicine for delivery, and circadian biology for temporal optimization, the core aims of constructing meaningful biomechanical models and effectively applying them in both education and clinical practice can be achieved with unprecedented precision and impact.

## 7 Conclusion

In conclusion, the integration of cellular biomechanics models into the analysis of neurological disease mechanisms and their subsequent application in educational practices underscores their core value in advancing our understanding of these complex disorders. The intricate interplay between cellular structures and biomechanical forces provides a unique lens through which we can examine the pathophysiology of neurological diseases. By elucidating the biomechanical underpinnings of cellular interactions, we can develop more accurate models that reflect the true nature of disease progression, thereby enhancing our diagnostic and therapeutic strategies.

Moreover, the translation of these models into educational settings highlights the importance of interdisciplinary collaboration among biomechanics, education, and clinical medicine. Such collaboration is essential for fostering innovation in neurological rehabilitation. For instance, by combining insights from biomechanics with pedagogical strategies, educators can create immersive learning experiences that engage students and deepen their understanding of neurological disorders. This approach not only enriches the curriculum but also prepares future healthcare professionals to approach neurological rehabilitation with a comprehensive and informed perspective.

The necessity of interdisciplinary cooperation is further emphasized by the complexity of neurological diseases, which often require multifaceted treatment approaches. By bridging the gap between basic science and clinical application, we can develop personalized rehabilitation programs that are informed by both biomechanical principles and clinical insights. This synergy is crucial for driving innovation in neurorehabilitation, as it allows for the design of targeted interventions that address the specific needs of patients.

In summary, the application of cellular biomechanics models in understanding neurological diseases and their integration into educational practices represent a significant advancement in both research and teaching. The collaborative efforts across disciplines will undoubtedly pave the way for novel approaches in neurological rehabilitation, ultimately improving patient outcomes and enhancing the quality of care in this challenging field.

## Data Availability

The original contributions presented in the study are included in the article/supplementary material, further inquiries can be directed to the corresponding author.
